# Concerns on the Growing Use of Lithium: The Pros and Cons

**DOI:** 10.5812/ircmj.13756

**Published:** 2013-08-05

**Authors:** Soodabeh Saeidnia, Mohammad Abdollahi

**Affiliations:** 1 Medicinal Plants Research Center, Faculty of Pharmacy, Tehran University of Medical Sciences, Tehran, IR Iran.; 2 Department of Toxicology and Pharmacology, Faculty of Pharmacy and Pharmaceutical Sciences Research Center, Tehran University of Medical Sciences, Tehran, IR Iran

**Keywords:** Lithium, Electrolytes, Toxicity, Environment

**Figure 1.**
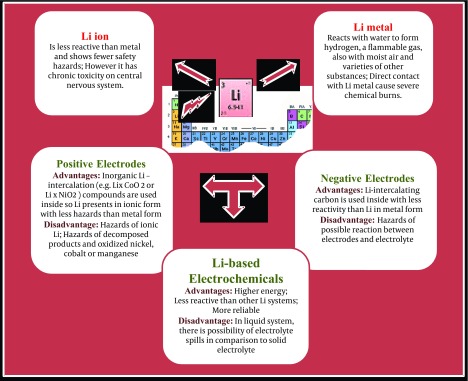
Advantages and Disadvantages of Various Electrochemical Systems Containing Lithium; Li Compounds are Applied in Both Negative and Positive Electrodes as Well as Electrolyte of Li ion Cells

The Greek word, lithos means stone that is the origin of a metal named “lithium” (Li), the 27 ^th ^most abundant, the lightest alkali, highly reactive and flammable metal within chemical elements. Li is never found freely in the nature due to high reactivity and easily found as a soluble ion in water, so that it is ordinary gained from brines and clays. But, Li is commercially obtained from a mixture of Li and potassium chlorides. The Li minerals with high commercial value are belonging to silicates, micas and phosphates. Lithium has low atomic mass and is observed rarely in the solar system than other chemical elements. In nuclear physics, Li-6 deuteride is employed as a fusion fuel in staged thermonuclear weapons (1, 2). Because Li is employed in the batteries with the greatest effect on metal depletion and occurring remarkable environmental and health impacts, here we aim to describe whether Li-based systems can release Li as a toxic metal into the nature resulting in accumulation inside plants, animals and finally human body and consequently any toxic effects. The Li ion based system is an advanced technology. Although managing the thermal production, and protecting the electrical and gases have been performed, environmental pollution of battery materials is still a concern (Figure 1).

**Figure 2.**
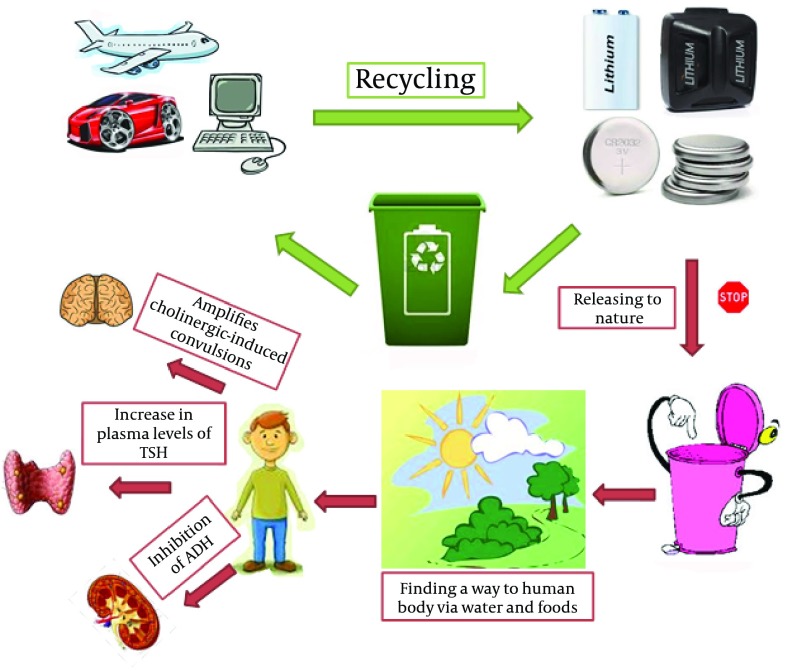
Recycling and/or Releasing the Lithium in the Nature and Accumulation in Human Body to Cause Damages in Diverse Organs

However, literature review gives estimation about toxicity to human (on the basis of per MegaJoule of capacity) as follows: Nickel-metal hydride and sodium-sulfur batteries< Li-ion batteries< nickel-cadmium batteries< lead-acid batteries. However, Li batteries has the lowest performance of greenhouse gases discharge, because up to 12.5 kg of CO2 equivalent discharged per one kg of Li batteries, while this performance is the best for sodium-sulfur and lead-acid batteries. Meanwhile, production of Li batteries wastes high energy. On the other side, battery disposal can cause adverse impacts on environmental and health safety on both human and wildlife especially where consumers get rid of batteries together with other trashes in the urban solid disposal materials (Figure 2) (3).

The Li requirement in human is assumed below 100 μg/day, even though it shows critically therapeutic activities such as stabilization of neuronal activities, neuro-protection and support of neuronal plasticity resulting in beneficial effects in mood-disorders therapy (4). Lithium salts are not considered very toxic except its hydrides, Li tetrahydroaluminate and Li tetrahydroborate. Taking together, Li is classified among the health, physiochemical and ecotoxicological hazards on the basis of the National Occupational Health and Safety Commission (NOHSC) approved criteria, of which Li itself, Li aluminium hydride, and Li methanolate are under the Danish list of dangerous compounds ( 5 , 6 ). Interestingly Li can even enter salivary glands where it causes kinds of adverse effects. A summary of possible toxicities of Li accumulation in human body are exhibited in Table 1.

**Table 1.** A Summary of Lithium Toxicity Due to Accumulation in Human Body (6-12)

Toxicity	Observations
**Genetic toxicity**	Causes disturbances in the development of invertebrates; Inhibition of development in whole rat embryos; Reduction of the numbers and weight of the litter and other abnormalities in intact animals; Creating malformations in the offspring of pregnant mice that received Li in high doses (6)
**Teratogenicity**	There is no association between Li treatment and accelerated teratogenic risk; Developing vascular system may be a target for lithium; It probably induce cell death in the neuroepithelium resulted in neural tube defects (6)
**Mutagenicity**	Binding selectively to DNA and competing with Mg2+; impairing DNA synthesis and repair; No effect has been observed with lithium chloride in strains of Bacillus aluminium and with trilithium citrate in the Ames test on Salmonella typhimurium, and in the sex-linked recessive lethality test in Drosophila melanogaster (6)
**Carcinogenicity**	There is no information on possible carcinogenic effects of Li (6)
**Environmental toxicity**	Li levels in surface waters were measured below 0.04 mg/L and can be increased in contaminated streams; Detecting at low concentrations 3 mg/L in the rivers; Seawater contains approximately 0.17 mg/L Li; Li levels in ground water is around 0.5 mg/L (6-8)
**Aquatic toxicity**	The acute environmental effect concentration ( EC50) on Daphnia magna is 33-197 mg/L (about 1000 times more than the level in fresh water); Both lithium chloride and sulfate are highly water soluble so that dissociate in aqueous environment; No lithium compounds are classified for adverse environmental effects and bioaccumulation (9)
**Renal toxicity**	Occurring histological alteration resulted in nephron inability to concentrate urine; Competing with sodium, potassium, magnesium, and calcium, then displacing them from intracellular and bone sites (10)
**Reproductive toxicity**	Passing the placenta; Accelerating the incidence of Ebstein’s anomaly in babies born from mothers who had Li therapy; There is low risk of fetal cardiovascular malformation; Reversible impotency in man (lowering sperm viability with no alteration in count or motility) (6, 11, 12)
**Neurotoxicity**	It may happen even at therapeutic plasma levels due to interaction with other drugs such as Maloteau; Hand tremor is a common sign; Irreversible neurotoxicity with cerebella abnormalities (6, 11)
**Hypothyroidism**	Enhancing the concentration of TSH together with accelerating the thyroid antibody titers in patients who had these antibodies before Li therapy; More common in female (6, 12)
**Gastrointestinal toxicity**	Vomiting and diarrhea may be primary symptoms of Li toxicity in patients with chronic exposure and therapy (6, 12)
**Salivary gland**	Affecting the secretory mechanisms of both submandibular and parotid glands not related to duration of treatment. Mostly through interference with the phosphoinositide cycle (13, 14)

The toxicity concern on Li batteries is more raised when this metal can be absorbed and accumulated by edible plants. As far as literature show, Li is able to take up by diverse plants; nevertheless it is not necessary for herbal growth and development. However it is reported that Li can stimulate the growth of some plants. The ordinary level of Li in plants is reported 0.2-30 ppm depending upon preferential uptake. Unfortunately, some edible and medicinal plants such as Cirsium arvense (trivial English name: Creeping Thistle) and Solanum dulcamera (trivial English name: bittersweet, bittersweet nightshade, nightshade or blue bindweed) are able to accumulate Li three to six times more than other plants. Carduus arvense (trivial English name: Canada or plume thistle) and Holoschoenus vulgaris (trivial English name: Roundhead Bulrush) may accumulate Li at levels between 99.6 to 226.4 mg/g. Li amount is reported from 0.01 ppm (on the basis of dried weight) in bananas to 55 ppm in oats. Interestingly, Li is almost toxic in Citrus plants (6).

Although Li is not considered as necessary element neither in animals nor in humans, it can be incorporated in the food chain from soils via flora to human. Minimizing the waste of Li batteries is definitely the most important way to reduce the possible effects of Li on environment and consequently its toxicity in human and animals. This might be attributed to the high levels of battery manufacturing and electrochemical systems containing Li. Certainly, recovering the useful metals inside batteries and recycling the plastic constituents are essential to reduce the releasing of Li into the nature and then its accumulation in human body. In fact, the safety of such batteries must enhance alongside the developing reliability and durability. However, much careful shipping is necessary for Li batteries, which contains Li-ion instead of Li-metal. Additionally, companies and manufacturers are able to choose which design for batteries needs fewer regulations in shipping term. Alongside the above comments, protection of the occupational safety can reduce the probable hazards of Li-ion batteries. For instance, workers might be exposed to Li via dust inhalation. Therefore, lowering dust dispersion and improving dust collection are two beneficial ways to decrease the Li exposure at workplace (12).

bitter In conclusion, although Li batteries production and application are the great hallmark of progress in advanced and developing countries, careless attention to dispose of such systems may lead to release of this toxic metal into the nature and consequently entrances to edible plants and animals and then accumulation in human body. Regarding the Li potential in causing toxicity in the central nervous system, gastrointestinal tract, reproductive and endocrine systems as well as genotoxicity, mutagenicity and teratogenicity, protection of the occupational safety and minimizing the waste of Li batteries are highly recommended to companies and manufacturers.
